# The unique histidine kinase, AtcS, regulates motility and pathogenicity of the periodontal pathobiont, *Treponema denticola*

**DOI:** 10.1128/iai.00112-25

**Published:** 2025-04-02

**Authors:** Doaa N. Abdallah, Annie N. Hinson, Aidan D. Moylan, Dhara T. Patel, Bin Zhu, Richard T. Marconi, Daniel P. Miller

**Affiliations:** 1 Department of Microbiology and Immunology, School of Medicine, Virginia Commonwealth University 542826, Richmond, Virginia, USA; 2 Massey Comprehensive Cancer Center, Virginia Commonwealth University 172856, Richmond, Virginia, USA; Stanford University School of Medicine, Stanford, California, USA

**Keywords:** *Treponema denticola*, periodontitis, two-component regulatory systems, *Treponema*, spirochetes, histidine kinase

## Abstract

*Treponema denticola* is an obligate colonizer of the human gingival crevice and, along with other pathobionts, is highly associated with the development of periodontal disease. As periodontal disease develops, significant environmental changes occur in the subgingival crevice and oral microbiome. The ability to sense and respond to changing environmental conditions is essential to the ability of *T. denticola* to thrive and cause disease. Yet, our understanding of *T. denticola* sensory transduction and gene regulatory mechanisms is nearly absent. The AtcSR two-component system has been predicted to regulate several cellular processes, but its role in *T. denticola* adaptive responses has not been investigated. To address this knowledge gap, we constructed a deletion of the *atcS* gene, encoding the histidine kinase. We performed RNA sequencing, demonstrating that the deletion of *atcS* results in significant changes in the transcriptome of *T. denticola*. Most notably, the transcription of genes encoding proteins involved in motility and the dentilisin protease complex was reduced. Consistent with this, the deletion mutant displayed reduced dentilisin activity and motility. These phenotypes are critical to interactions with host cells and the pathogenicity of *T. denticola*. This aligns with our observation that the *atcS*-deficient strain had attenuated attachment and invasion of gingival epithelial cells and failed to induce alveolar bone loss in a murine periodontitis model, processes that are central to *T. denticola* virulence. This study is a significant step toward defining the role of the AtcSR two-component system in *T. denticola* pathogenicity.

## INTRODUCTION

Periodontal disease destroys tooth-supporting structures due to chronic inflammation induced by a dysbiotic microbiome. Periodontitis affects nearly half of adults 30 years or older in the United States, and more than 10% of the adult population worldwide has stage IV disease, resulting in edentulism and reduced quality of life (1, 2). Individuals with periodontitis are at increased risk of developing systemic diseases such as cardiovascular disease, type II diabetes, and gastrointestinal or head and neck cancers (3–6). The incidence and chronic nature of the disease contribute to it being one of the most expensive diseases to treat (>$350 billion globally), trailing only heart disease and diabetes mellitus (7, 8).

Periodontitis results from a microbial shift from largely gram-positive, facultative organisms to a more diverse dysbiotic community composed predominantly of gram-negative bacteria and spirochetes that are proteolytic and less tolerant of oxygen (9, 10). *Treponema denticola* is an atypical gram-negative, anaerobic spirochete that localizes to the deepest region of the subgingival sulcus (11). *T. denticola,* a member of Socranky’s “Red Complex,” is a periodontal pathobiont whose incidence and abundance are associated with periodontitis (12–15). In the gingival sulcus, *T. denticola* localizes at the leading edge of the plaque, loosely adherent to the biofilm and in direct contact with the junctional epithelium. *T. denticola* can attach to and invade gingival keratinocytes, fibroblasts, and periodontal ligament cells, enabling it to penetrate gingival tissues (summarized by Dashper et al. [16]). These interactions drive pro-inflammatory responses central to periodontitis development and associated with oral cancers (17, 18).

The surrounding ecology shapes the biogeography of the plaque biofilm, while the dysbiotic biofilm can also alter the environment to make it more habitable for strict anaerobes (19, 20). To thrive, *T. denticola* must sense and adapt to its dynamic environment. The molecular signaling strategies of *T. denticola* are poorly characterized, but it utilizes several two-component systems (TCSs) and the nucleotide secondary messengers c-di-GMP and c-di-AMP (21–25). The most abundant and best-characterized signaling systems in bacteria are the TCSs, which typically sense environmental stimuli to regulate gene expression or enzyme activity to respond to their environment physiologically (26). The canonical TCS comprises a membrane-bound or cytoplasmic histidine kinase and a response regulator. Histidine kinases typically harbor one of many sensing domains that influence autophosphorylation and the subsequent phosphoryl transfer to an Asp residue on the response regulator, which impacts the activity of an output domain (i.e., helix-turn-helix DNA-binding domain, protein–protein interactions, or enzymatic activity such as diguanylate cyclases). The *T. denticola* ATCC 35405 genome encodes five complete TCS, four orphan histidine kinases and response regulators, and a single hybrid TCS, where a single protein performs the functions of both the histidine kinase and the response regulator (27). Despite their importance in bacterial physiology and pathogenesis, our understanding of TCS in *T. denticola* is undeveloped.

AtcS (histidine kinase; TDE0032) and AtcR (response regulator; TDE0033) form a functional TCS whose expression is upregulated during the late-logarithmic and early-stationary phase of growth (21). AtcS contains a transmembrane domain with no predicted sensing domain, leaving the nature of its regulation or stimulation enigmatic. AtcR contains a LytTR DNA-binding domain, the only such domain in spirochetes. An earlier study that employed electrophoretic mobility shift assays (EMSAs) and bioinformatic analyses provided indirect evidence that AtcR regulates motility-associated genes, an ATP-binding cassette (ABC)-type transporter, and the only other TCS of *T. denticola* that has been characterized to date (Hpk2/Rrp2) (28). Hpk2 (histidine kinase; TDE1970) and Rrp2 (response regulator; TDE1969) also form a functional TCS that is growth-phase regulated (25). Interestingly, Hpk2 contains a Per-Arnt-Sim (PAS) domain that senses oxygen as Hpk2 autophosphorylation is enhanced under anaerobic conditions (29). Rrp2 remains unstudied but is predicted to be a sigma-54 enhancer-binding protein.

Beyond signal transduction, *T. denticola* relies on other virulence factors to acquire nutrients, evade the immune response, and establish infection. Two *T. denticola* outer sheath protein complexes are essential for host-cell interactions and pathogenicity. Dentilisin is a chymotrypsin-like protease complex composed of PrtP and the accessory proteins PrcA and PrcB (30, 31). Collateral damage to its role in nutrient acquisition, dentilisin cleaves many host proteins such as complement proteins, cytokines, and extracellular matrix (ECM) components (32). Dentilisin is also required for proper processing and surface presentation of the major sheath protein (Msp), the other dominant oligomeric protein complex (33, 34). Msp is critical to *T. denticola*’s interactions with host cells and tissue as it binds to fibronectin, fibrinogen, laminin, and plasminogen (32). Msp is a pore-forming protein that is cytotoxic and disruptive to host actin filament processing, which impacts migration (35).

In this study, we generated a *T. denticola* strain 35405 *atcS* deletion mutant (Δ*atcS*) to better define the role of AtcS in *T. denticola* physiology and pathogenesis. The transcriptome of the Δ*atcS* mutant was determined using RNA seq. We found the Δ*atcS* strain had reduced motility compared to the parental strain, which was consistent with the reduced expression of motility-associated genes. We found a reduced movement toward glucose and hemin as chemoattractants. However, this phenotype is likely more driven by the reduced motility than a chemotactic defect. We also found a reduced gene expression that encodes for the dentilisin complex, and the Δ*atcS* strain had attenuated dentilisin activity. As dentilisin is vital for interactions with host cells, we demonstrated the Δ*atcS* strain had reduced attachment to and invasion of gingival keratinocytes. Finally, the Δ*atcS* strain failed to induce alveolar bone loss in a murine periodontitis model, while *T. denticola* ATCC 35405 induced significant bone resorption, suggesting signaling via AtcS contributes to pathogenesis. Collectively, this study represents a significant advancement in our understanding of molecular signaling mechanisms in *T. denticola* by demonstrating the physiological role of AtcS.

## RESULTS

### Deletion of *atcS* results in a minor growth defect

**Fig 1:**
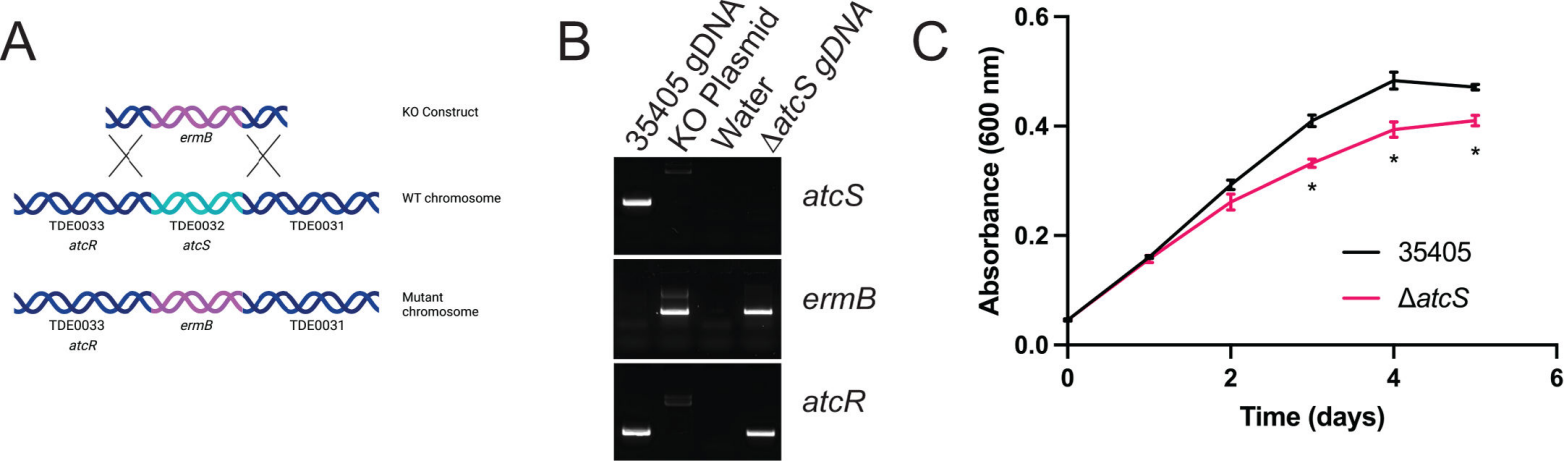
Deletion of *atcS* (*TDE0032*) results in a minor growth defect. (**A**) The *atcS* gene was replaced by *ermB* by allelic exchange. (**B**) The knockout was confirmed by PCR using primers specific to *atcS* and *ermB*. We confirmed the deletion of *atcS* did not impact the presence of the upstream *atcR* gene. (**C**) We monitored the growth of Δ*atcS* compared to the parental ATCC 35405 by measuring the absorbance at 600 nm daily. Data are the average of three independent cultures with standard error of the mean (three replicate measurements per time point). Growth differences were assessed by a Student’s *t*-test on each day of growth (*P < 0.001).

Previous studies described the molecular details of AtcS autophosphorylation and transfer to AtcR. To understand how AtcS signaling impacts *T. denticola* physiology, we constructed a *tde0032* knockout by allelic exchange in the *T. denticola* type strain ATCC 35405 (Fig. 1A). Using a combination of PCR and qRT-PCR, we confirmed the loss of the *atcS* gene without impacting the expression of *atcR* or *tde0034*, two genes co-transcribed in the operon (Fig. 1B; Fig. S1). We also monitored the growth of the Δ*atcS* strain compared to the parental strain in new oral spirochete (NOS) broth by absorbance at 600 nm. The growth of the Δ*atcS* strain mirrored ATCC 35405 for the first 48 hours but displayed a ~20% reduction in growth starting at day 3 and continuing to day 5 (Fig. 1C; Fig. S1). While we did not anticipate AtcS would impact the *in vitro* growth of *T. denticola*, these results are consistent with the observation that *atcS* expression and protein abundance are elevated at 4 days of growth compared to 2 days (21). Collectively, these results demonstrate that AtcS is vital for maintaining the fitness of *T. denticola in vitro*.

### Transcriptomic changes resulting from the deletion of *atcS*

**Fig 2:**
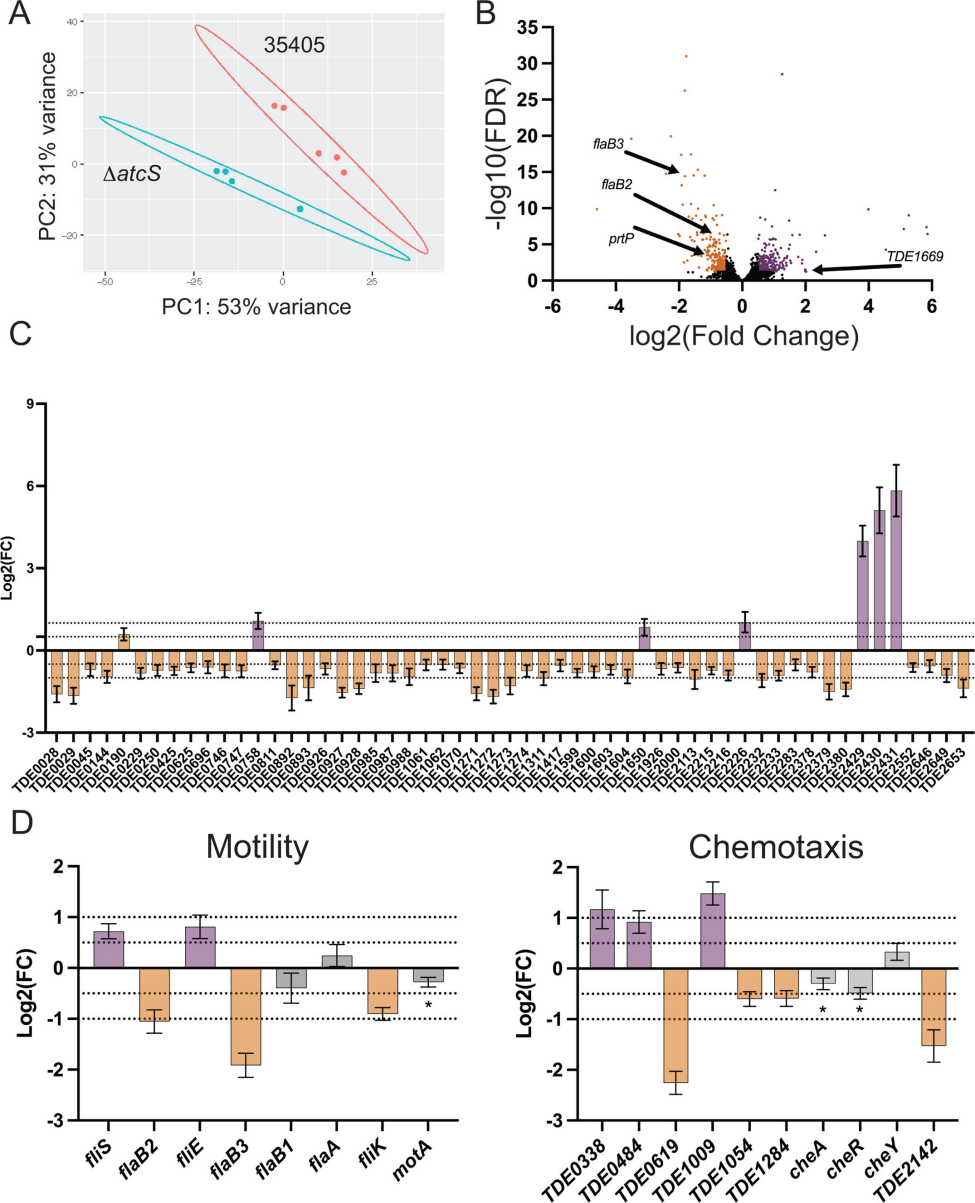
RNA seq revealed significant differences in transport systems and motility-associated genes. (**A**) Principal component analysis shows tight clustering of the ATCC 35405 and Δ*atcS* samples (*n* = 5). (**B**) Scatter plot of differentially expressed genes (±0.5 log2 [fold change]; q-value < 0.05). Purple circles represent upregulated genes, downregulated genes are orange, and genes with no differential expression are black. (**C and D**) Graphs of differentially expressed genes associated with transport systems, motility, and chemotaxis, respectively. Data are the log2FC with standard error. Dashed lines represent cutoffs for the relaxed and stringent analyses. Genes significantly repressed in Δ*atcS* relative to ATCC 35405 are orange, while induced genes are purple. Gray bars were not beyond the ±0.5 log2 (fold change) for significance, but in some cases, the q-value was below the threshold for statistical significance (*q-value < 0.05).

We sought to understand how AtcS may impact the physiology of *T. denticola* by comparing the transcriptome of the Δ*atcS* strain to ATCC 35405. We grew both strains in NOS for 4 days before collecting RNA for RNA seq. We identified 449 differentially expressed genes (±0.5 log2 fold change [log2FC], <0.05 false discovery rate), representing 17.7% of the protein-coding genes between Δ*atcS* and ATCC 35405 (Fig. 2). Using more rigorous cutoffs (±1.0 log2 fold change, <0.01 false discovery rate), 104 genes were differentially expressed (Table S1). We discuss the results using the relaxed analysis to be more inclusive. The differential expression of all genes is available in Table S1. Transcriptional changes were balanced between induction (218 genes were upregulated) and repression (231 genes were downregulated). As expected, no reads were mapped to *atcS* in the knockout, and the expression of all other genes in the *atcS*-containing operon was unchanged. A vital consideration in the Δ*atcS* strain is the potential for transcriptional regulation beyond the AtcS/R signaling system. We observed a significant differential expression of 14 putative transcriptional regulators, including the orphan response regulator *TDE2324* (Fig. S2). We also observed the downregulation of an uncharacterized histidine kinase (*TDE0656*; −0.57 log2FC), although the expression of the response regulator (*TDE0655*; −0.22 log2FC) was reduced, it was not significant. Finally, we also observed the upregulation of two putative c-di-GMP phosphodiesterases (*TDE0128*; 0.67 log2FC and *TDE2302*; 0.73 log2FC), suggesting a possible impact on c-di-GMP signaling in the Δ*atcS* strain.

Strikingly, the most abundant functional class of differentially expressed genes is in transport systems (Fig. 2C). ATP-binding cassette (ABC) transporters were overwhelmingly downregulated; 46 genes associated with 26 different ABC transport systems were downregulated, while only four ABC transport-associated genes were upregulated. While the overall trend was a reduced expression of ABC transport systems, all the genes within an operon from *TDE2429* to *TDE2431* were among the most upregulated genes in the Δ*atcS* strain. In addition to ABC-type transport systems, *TDE0190* encodes a putative drug/metabolite transporter that is upregulated, and *TDE0250* encodes a putative sodium-dependent transporter that is downregulated. While most of these transporters’ functions remain unknown, these data suggest that deletion of *atcS* significantly disrupts solute and metabolite transport.

Another striking observation from the RNA-seq analysis is the aberrant regulation of motility- and chemotaxis-associated genes (Fig. 2D; Fig. S3). Again, most of the differentially expressed motility-associated genes were downregulated. *TDE2770* (*fliK*), the first gene in the operon (*TDE2760-TDE2770*) encoding most flagellar hook and motor proteins, was downregulated but was the only differentially expressed gene in the operon. The flagellar structural genes *flaB2* (*TDE1004*) and *flaB3* (*TDE1475*) were significantly downregulated, while *flaB1* (*TDE1477*) and *flaA* (*TDE1712*) were not differentially expressed. Seven putative chemotaxis-associated genes were differentially expressed (three upregulated and four downregulated).

### Impact of AtcS on motility and chemotaxis of *T. denticola*

**Fig 3:**
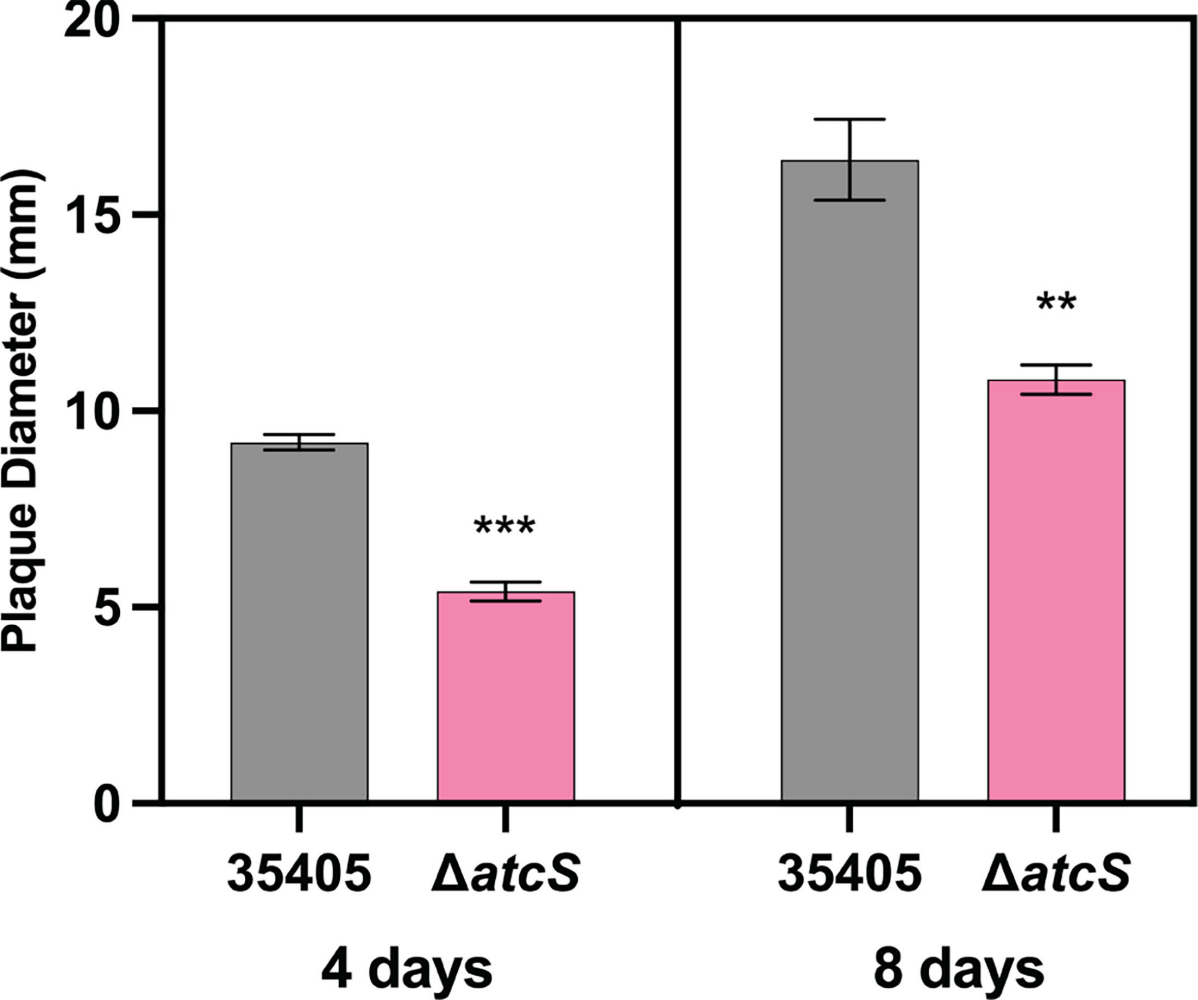
The deletion of *atcS* attenuated *T. denticola* motility. Wild type (black) and Δ*atcS* (pink) were inoculated into NOS plates solidified with Noble agar, and swarming motility was monitored by measuring the plaque diameter at 4 and 8 days of incubation. Data are the average of five independent experiments with standard deviation and were analyzed using the Student’s *t*-test (**P < 0.01, ***P < 0.001).

**Fig 4:**
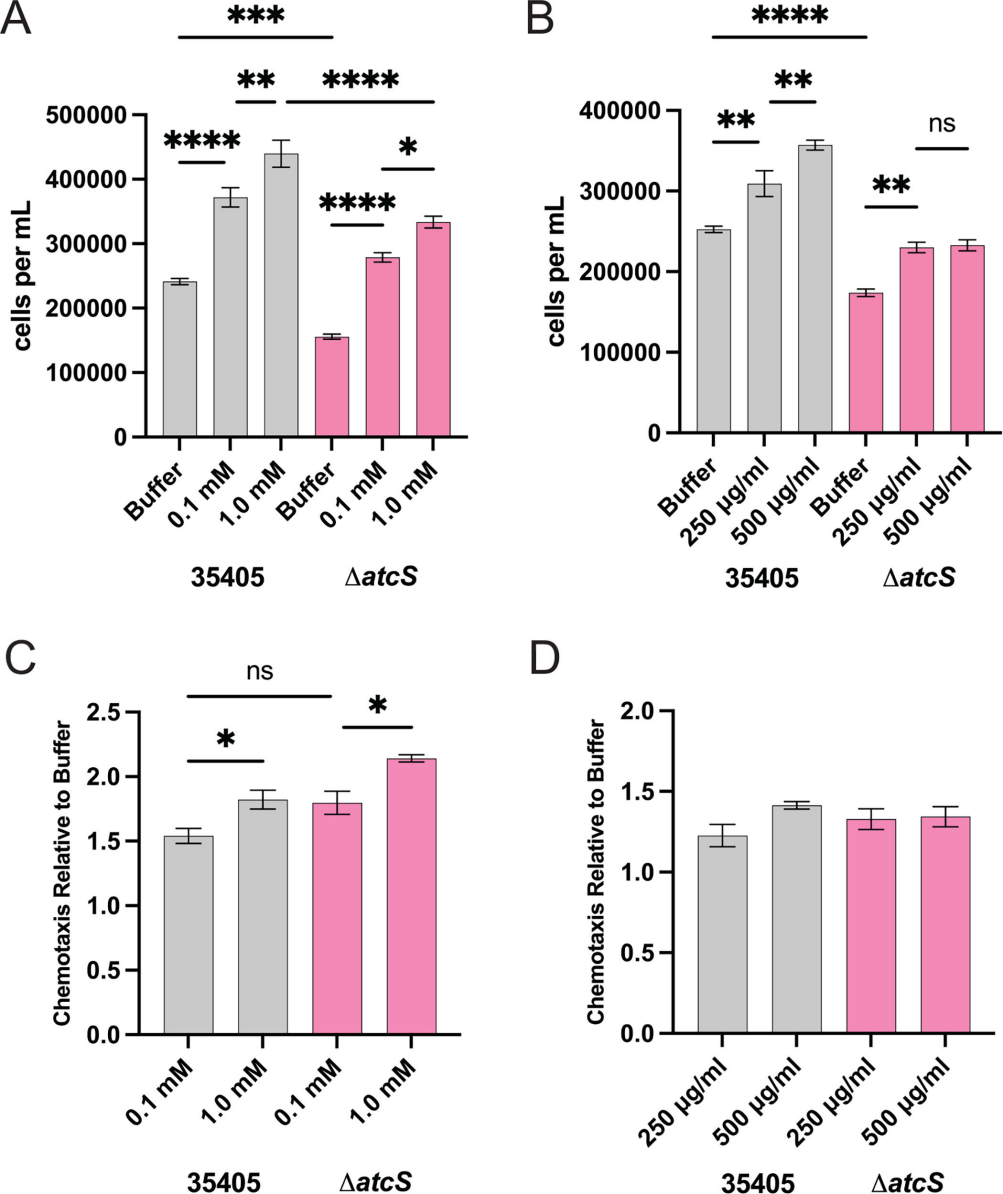
AtcS is not required for chemotactic response to glucose or hemin. The number of spirochetes per milliliter for ATCC 35405 (black) and Δ*atcS* (pink) was assessed by dark-field microscopy after treatment with either 0.1 and 1.0 mM glucose (**A**) or 250 and 500 µg/mL hemin (**B**) after 3 hours. To control for known differences in motility, the chemotactic response for both glucose (**C**) and hemin (**D**) was normalized to buffer alone. Data are the average of three independent experiments with standard error and were analyzed by one-way ANOVA with Tukey’s post hoc test (*P < 0.05, **P < 0.01, ***P < 0.001, ****P < 0.0001). ns, not significant.

Our transcriptomic analysis suggested that motility and chemotaxis may be altered in the Δ*atcS* mutant. We first sought to characterize swarming motility within semi-solid agar. A bacterial suspension of *T. denticola* ATCC 35405 and Δ*atcS* was placed in NOS plates containing 0.5% Noble agar and was incubated for either 2 or 4 days before measuring swarming diameters (Fig. 3; Fig. S3). We observed significantly reduced plaque diameters between the Δ*atcS* and wild-type ATCC 35405 on days 4 and 8. We then sought to characterize chemotactic responses to glucose and hemin, known chemoattractants (36, 37). We observed a dose-dependent migration toward glucose and hemin for ATCC 35405, and bacterial movement toward glucose and hemin for Δ*atcS*, albeit at a reduced level compared to ATCC 35405 (Fig. 4A and B). To account for the inherent difference in motility, we normalized migration to glucose and hemin to the buffer control condition (Fig. 4C and D). Here, we observed no significant difference in chemotaxis between 35405 and Δ*atcS*, suggesting the difference in absolute cell numbers in the capillary tubes is due to reduced motility and not attenuated chemotactic responses. It is also worth noting that both swarming and swimming motility were reduced using these complementary approaches.

### Δ*atcS* has reduced expression of dentilisin complex genes and attenuated proteolytic activity

**Fig 5:**
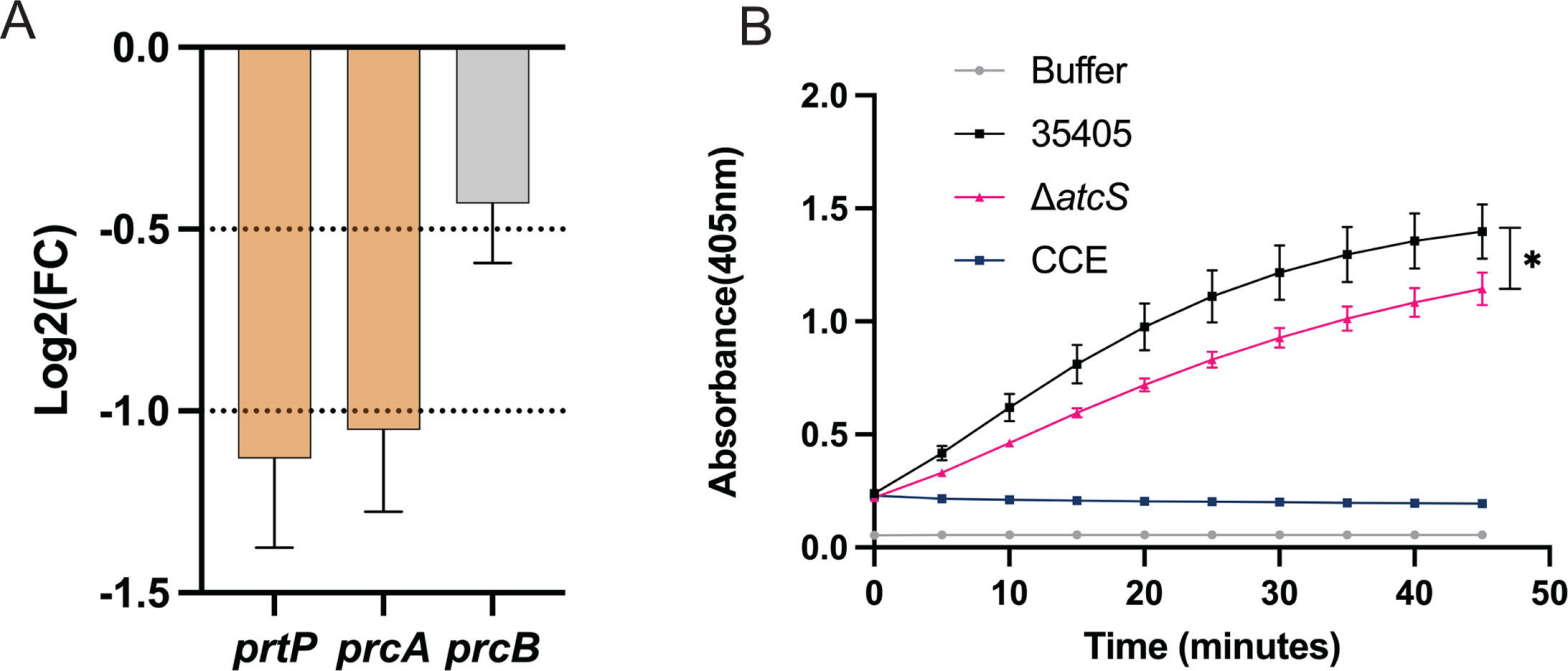
The Δ*atcS* strain has reduced surface PrtP and dentilisin activity. (**A**) Graph of the expression of dentilisin complex genes in the RNA seq. Data are the log2FC with standard error. Dashed lines represent cutoffs for the relaxed and stringent analyses. Genes significantly repressed in Δ*atcS* relative to ATCC 35405 are orange. (**B**) The dentilisin activity of each strain was assessed by the hydrolysis of the SAAPFNA substrate colorimetrically by measuring the absorbance at 405 nm every 5 minutes for 45 minutes. Buffer alone and CCE were used as negative controls. Results are the average of three replicates with standard deviation. All time points from 10 to 45 minutes were significantly different according to a Student’s *t*-test for each time point (*P < 0.05). The graph shown is representative of three independent experiments.

The RNA-seq and qRT-PCR analysis revealed a significantly reduced expression of the dentilisin complex genes (*prtP*, *prcA*, and *prcB*; Fig. 5A; Fig. S4). Next, we sought to determine if reduced expression and PrtP protein influenced the dentilisin activity of Δ*atcS*. We monitored the cleavage of the synthetic, colorimetric chymotrypsin substrate succinyl-L-alanyl-L-alanyl-L-prolyl-L-phenylalanine-p-nitroanilide (SAAPFNA) (Fig. 5C; Fig. S4). The dentilisin-deficient strain, CCE, was used as a negative control along with PBS. Neither PBS nor CCE yielded any detectable cleavage of SAAPFNA, as expected. We observed a ~25% reduction in the dentilisin activity of Δ*atcS* compared to ATCC 35405. While the SAAPFNA cleavage between the two strains was statistically significant at all time points, this likely represents a modest reduction in dentilisin activity that may not significantly alter the biological role of dentilisin.

### *T. denticola* interactions with gingival epithelial cells involved AtcS signaling

**Fig 6:**
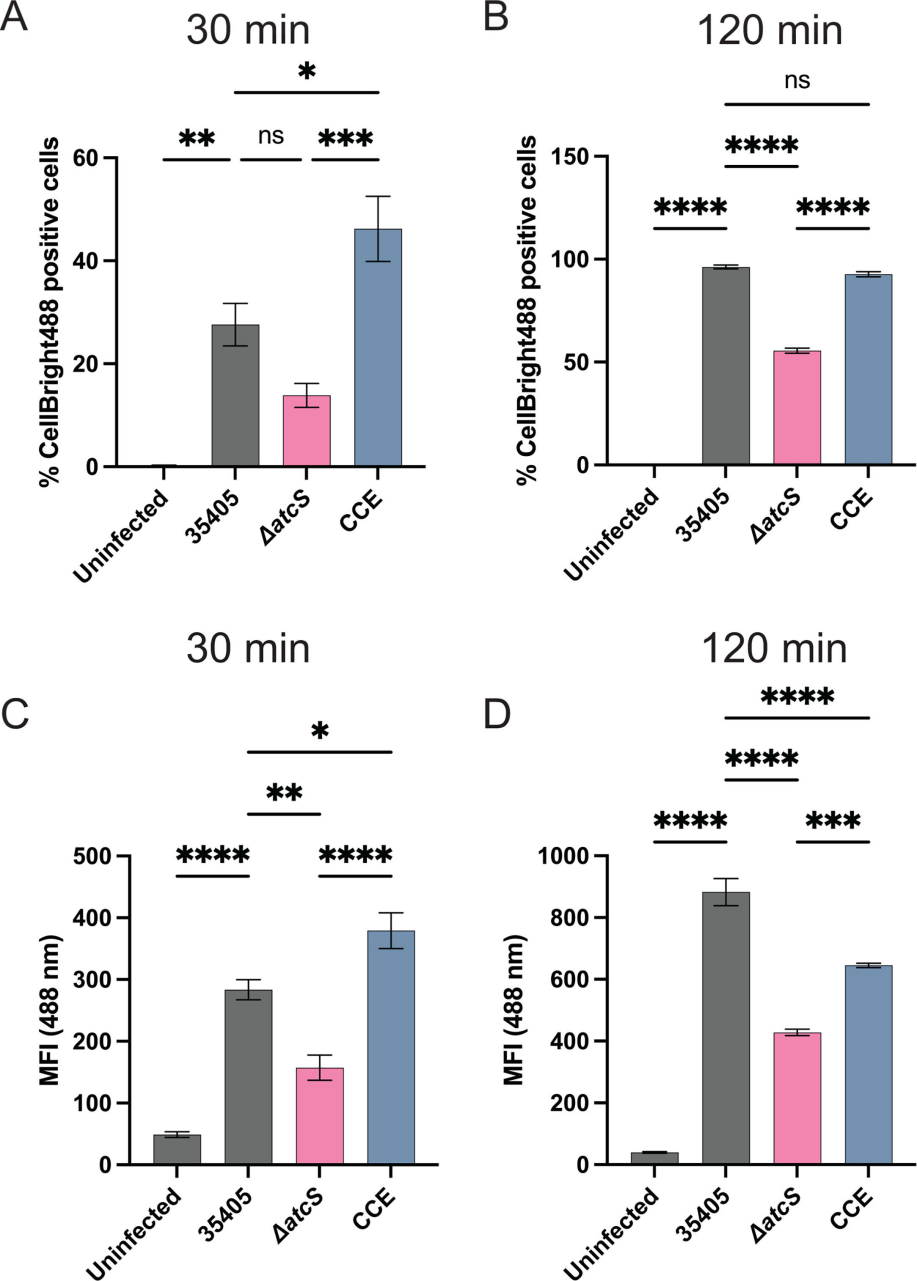
Deletion of *atcS* reduced adherence to TIGK cells. *T. denticola* strains were labeled with CellBrite-488 (Biotium). Flow cytometry assessed interactions with TIGK cells (MOI 100) after 30 and 120 minutes. Data are the average percentage of CellBrite-488-positive TIGK cells (**A and B**) or the mean fluorescent intensity (**C and D**) with standard error of the mean. Data are the average of four independent experiments with standard error of the mean. Each experiment counted roughly 10,000 TIGK cells. Data were analyzed by one-way ANOVA with Tukey’s post hoc test (*P < 0.05, **P < 0.01, ***P < 0.001, ****P < 0.0001). ns, not significant.

Dentilisin, Msp, and motility are involved in the attachment and invasion of host cells (38, 39). As the Δ*atcS* strain had reduced motility and dentilisin expression, we hypothesized that this strain would have reduced ability to engage with gingival keratinocytes. We used flow cytometry to assess *T. denticola* strains labeled with CellBrite-488 fluorescent membrane stain binding to telomerase immortalized gingival keratinocytes (TIGKs) (40) cells after 30 and 120 minutes (Fig. 6). After 30 minutes, we observed that 27.6 ± 4.1% (283.5 ± 16.2 mean fluorescent intensity [MFI]) of TIGKs were infected with ATCC 35405, while only 13.9 ± 2.3% (157.3 ± 20.3 MFI) of TIGKs were positive for Δ*atcS* infection. These trends were exacerbated after 120 minutes of infection with a 50% reduction in the percentage of infected TIGKs and MFI when infected with Δ*atcS* compared to ATCC 35405. Interestingly, infection with the dentilisin-deficient strain (CCE) resulted in more infected TIGKs after 30 minutes (46.2 ± 6.3%; 379 ± 28.9 MFI) compared to the ATCC 35405 strain. By 120 minutes post-infection, the percentage of infected TIGKs with CCE was nearly identical to ATCC 35405 but with a 27% reduction in MFI.

**Fig 7:**
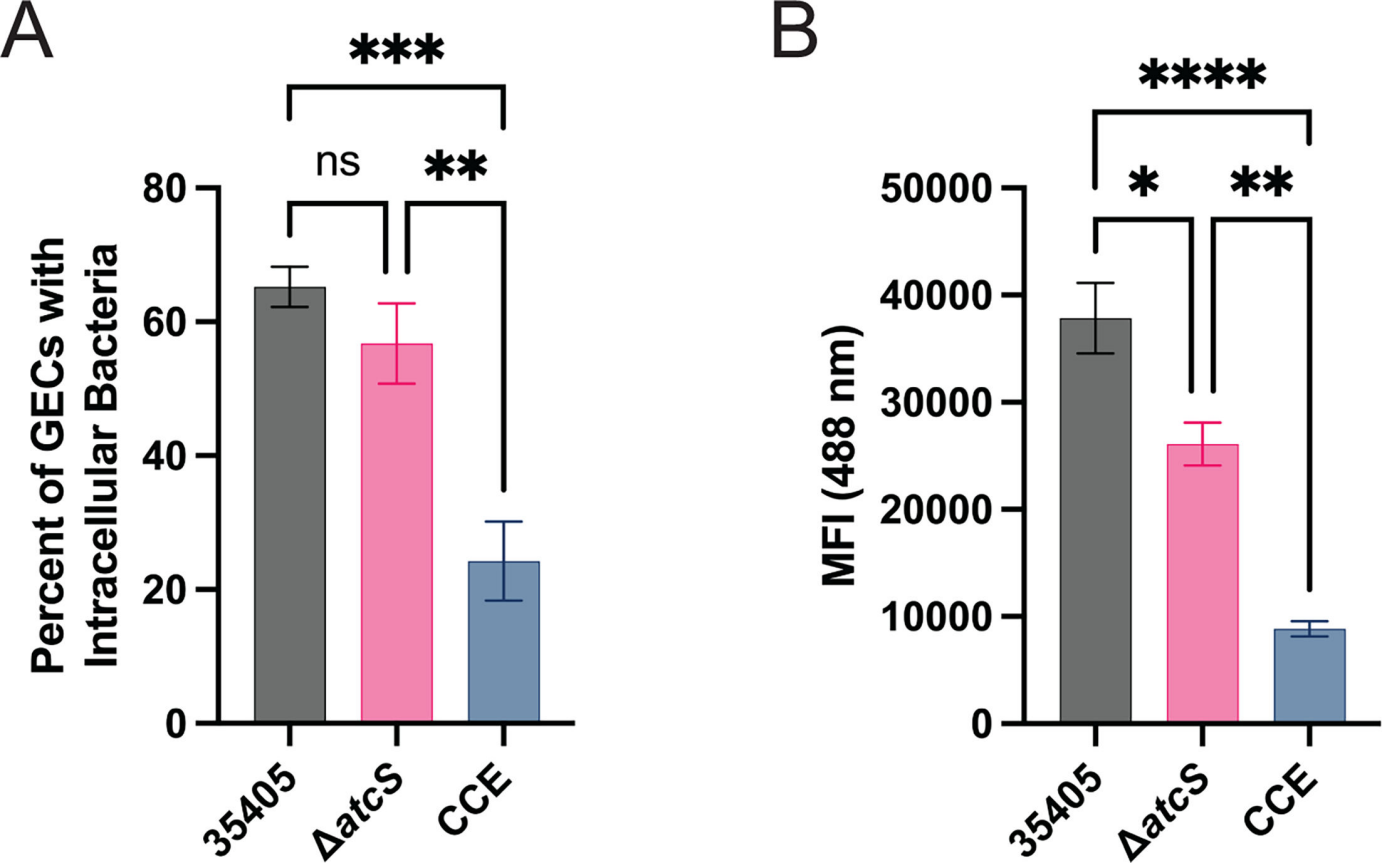
The Δ*atcS* strain is less invasive than wild-type *T. denticola*. *T. denticola* strains were labeled with CellBrite-488 (Biotium) before infecting TIGK cells. After the infection, TIGK cell membranes were labeled with CellBrite-640. Cells were analyzed using the Amnis ImageStreamX MkII to identify the TIGK plasma membrane and quantify the intracellular spirochetes. Data are the average percentage of TIGK cells with intracellular *T. denticola* (**A**) or the mean fluorescent intensity (**B**) with standard error of the mean (*n* = 4). Each experiment counted roughly 2,000 TIGK cells. Data were analyzed by one-way ANOVA with Tukey’s post hoc test (ns, not significant; *P < 0.05; **P < 0.01; ***P < 0.001; ****P < 0.0001).

We then sought to specifically investigate if *atcS* impacts the ability of *T. denticola* to invade TIGK cells using a Cytek ImageStream (Fig. 7). After 120 minutes of infection, we again observed fewer TIGK cells were positive for the Δ*atcS* strain than wild type. However, when we examined only the 488-positive TIGKs, we found no significant change in the amount of invaded *T. denticola* between the *atcS* knockout and the parental strain. In contrast, CCE had a more modest defect in adherence to TIGKs but a significant defect in the ability to invade within TIGK cells compared to ATCC 35405 (Fig. 6 and 7). Collectively, these data suggest the Δ*atcS* strain had reduced infectivity in a TIGK cell model, and this reduction is, at best, only partially explained by reduced dentilisin in the Δ*atcS* mutant. The defect is limited primarily to attachment, as when Δ*atcS* attaches to TIGKs, there is little to no difference in the invaded bacteria.

**Fig 8:**
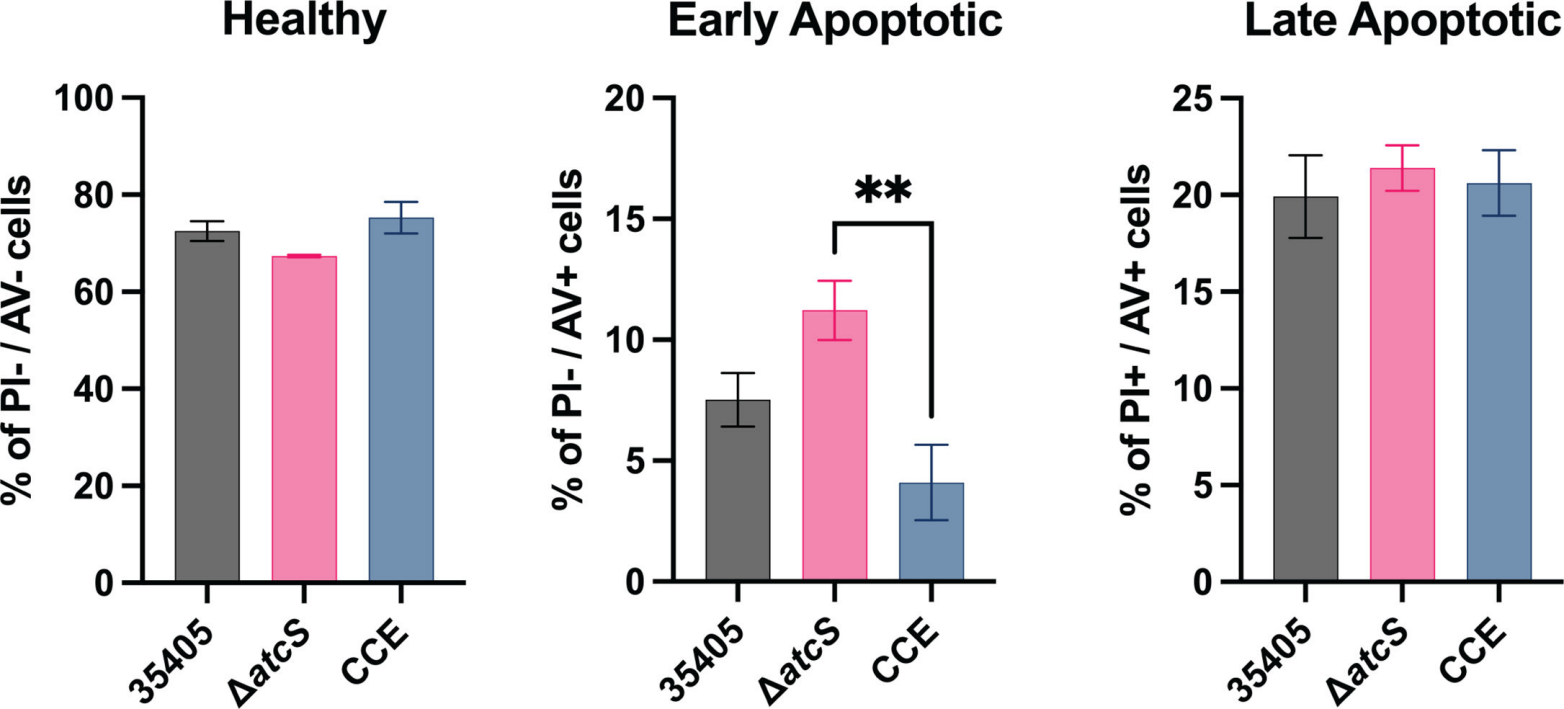
Neither AtcS nor PrtP contributes significantly to *T. denticola*-induced apoptosis of TIGK cells. TIGK cells were infected with *T. denticola* ATCC 35405, Δ*atcS*, or CCE at an MOI of 100 for 18 hours before the cells were stained with allophycocyanin (APC)-conjugated AV and PI and analyzed by flow cytometry using a BD Canto II. Cells were healthy if the staining was double-negative staining for PI and AV; early apoptotic cells were identified as AV positive but PI negative, and late apoptotic/necrotic cells were identified by double-positive staining for PI and AV. Results are the average of four independent experiments (each experiment counted ~10,000 cells) with SEM and were analyzed using a one-way ANOVA with Tukey’s post hoc test (**P < 0.01; otherwise, all groups were not significantly different).

Dentilisin activity promotes apoptosis in periodontal ligament cells (41). The deletion of *atcS* reduced the abundance and activity of dentilisin, which we expected to minimize *T. denticola*-induced apoptosis. However, cystalysin (*TDE1669*), which catalyzes the hydrolysis of L-cysteine to pyruvate, ammonia, and H_2_S, is upregulated in the Δ*atcS* strain and may be expected to increase apoptosis (42). As such, we sought to determine if the deletion of *atcS* impacts *T. denticola*-mediated apoptosis. We infected TIGK cells with *T. denticola* ATCC 35405, Δ*atcS*, or CCE for 18 hours at an MOI of 100. Cells were examined for apoptosis by staining with propidium iodide (PI) and Annexin V (AV), and quantified by flow cytometry (Fig. 8). Previous reports vary in the degree to which *T. denticola* induces apoptosis in various cell culture models. Using the TIGK cells, we observed that 70%–75% of TIGK cells remained healthy, and ~20% were late apoptotic or necrotic after 16 hours of infection with all *T. denticola* strains. There was no meaningful difference in early apoptotic cells when cells were infected with either CCE or Δ*atcS* compared to ATCC 35405. We did observe a significant difference between CCE and Δ*atcS*. These studies demonstrate that AtcS-dependent signaling contributes to attachment to gingival epithelial cells but does not impact invasion, nor does it influence the induction of apoptosis in TIGK cells.

### AtcS contributes to pathogenicity in a murine alveolar bone loss model

**Fig 9:**
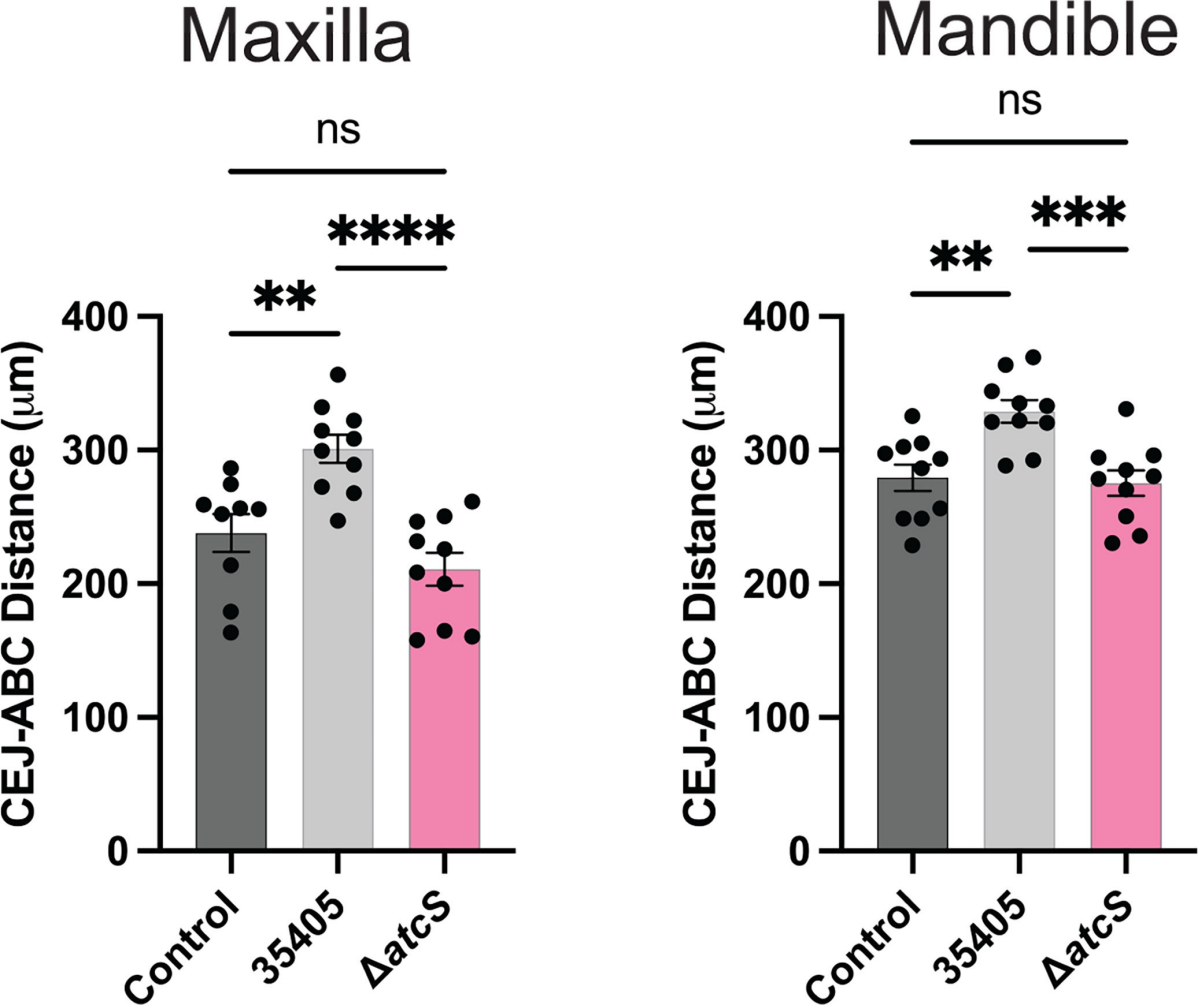
AtcS is required for *T. denticola* to induce alveolar bone loss. Female BALB/c mice were inoculated with either ATCC 35405 (black), Δ*atcS* (pink), or a vehicle control (gray) (*n* = 10 animals per group). Following 6 weeks of incubation, alveolar bone loss was assessed at the first maxillary and mandibular molar using μCT. Data are the average distance (μm) between the CEJ and the alveolar bone crest (ABC) for each mouse and the standard error of the mean. Data were analyzed by one-way ANOVA with Tukey’s post hoc test (**P < 0.01, ***P < 0.001, ****P < 0.0001). ns, not significant.

The collective disruption in motility, dentilisin activity, and interactions with gingival epithelial cells (GECs) suggested the Δ*atcS* strain may be less virulent than the parental ATCC 35405. We used a murine alveolar bone loss model to assess the role of AtcS in the pathogenicity of *T. denticola*. Ten BALB/c mice were orally infected with *T. denticola* ATCC 35405 or Δ*atcS* suspended in PBS with carboxymethylcellulose (CMC) or CMC/PBS only as a vehicle control. Following the infection and 6 weeks of incubation, alveolar bone loss was assessed by microcomputed tomography (μCT) by measuring the distance between the alveolar bone crest (ABC) and the cementoenamel junction (CEJ). Wild-type *T. denticola* induced significant alveolar bone loss by examining the first maxillary and mandibular molars. At the same time, Δ*atcS* had no change in the distance between the CEJ and ABC compared to the control (Fig. 9). When examining either the second maxillary or mandibular molar, we did not observe a statistically significant alveolar bone loss induced by ATCC 35405 compared to the vehicle control. Still, in both cases, the Δ*atcS* strain had significantly less distance between the CEJ and ABC than the wild type (Fig. S5). Collectively, these data demonstrate that AtcS is required for *T. denticola*-mediated alveolar bone loss in a murine periodontitis model.

## DISCUSSION

The *T. denticola* proteins AtcS and AtcR form a unique TCS only present in *T. denticola* and the closely related *T. putidum* (43). AtcS is a membrane-bound histidine kinase with no identifiable or conserved sensing domain. Only 2%–3% of histidine kinases lack a conserved sensing domain (44). The sensory function of these atypical proteins is thought to be within the transmembrane domain, either through interactions with other membrane proteins or conformation changes resulting from changes in the membrane microenvironment. The best-characterized example is the BceS kinase in *Bacillus subtilis*, which interacts with the BceAB ABC-transporter (45–47). When the antimicrobial peptide bacitracin is present, BceB binds bacitracin, causing a conformational change that allows for the transmembrane domains of BceB and BceS to interact, activating BceS kinase activity and subsequent signaling through BceR that regulates the expression of BceAB. The signals that influence AtcS activity remain unstudied, but it may be that AtcS signaling mimics the sensing strategy of BceS. This study showed significant dysregulation in ABC-transporter expression in the *atcS* mutant compared to ATCC 35405 (Fig. 2C). An ABC-transporter (TDE0028-0029) is adjacent to the *atcS*/*atcR* encoding operon and is negatively regulated in the *atcS*-deficient strain. Overall, we found that the deletion of *atcS* generally results in the downregulation of ABC-type transport systems. The major exception is an operon composed of *TDE2429-2431. TDE2431* encodes a putative bacteriocin-secreting ABC transporter, *TDE2430* encodes a putative HlyD-like adaptor protein, and *TDE2429* encodes a putative TolC protein. To date, the function of both these transport systems still needs to be studied. Future studies on these systems may enhance our understanding of *T. denticola* physiology and elucidate a mechanism that impacts AtcS activation.

AtcR is also an uncommon response regulator, as it is the only protein in spirochetes that contains a LytTR-DNA-binding domain. Our previous study identified a putative AtcR regulon using an iterative combination of bioinformatics and EMSAs (28). Despite extensive attempts, we have yet to knockout *atcR* (*TDE0033*), suggesting it may be inherently essential. Another explanation is a TDEII methylation site 76 bp downstream of the *atcR* gene, which may significantly limit the efficiency of homologous recombination (48). A limitation of our current study is that the results are mainly descriptive, and mechanistically demonstrating a direct regulation of gene expression by AtcR remains to be seen. While the differential expression of multiple transcriptional regulators confounds the results of this study, genes previously predicted to be regulated by AtcR were, in part, differentially expressed in the Δ*atcS* strain. AtcR bound to the promoters upstream of *TDE1210*, *TDE2770*, *TDE2220*, and *TDE1132*, and all four genes were differentially expressed in the Δ*atcS* (28). However, the expression of *TDE1971*, *TDE1514*, and *TDE2496* was not significantly different in this study despite demonstrating that AtcR binds to promoter regions upstream of these genes. It may be that other transcription factors, two-component, or secondary messaging systems also act on the expression of genes regulated by AtcR, which may explain why we do not observe a perfect correlation between our results.

Additionally, the phenotypes described in this study do not explain the modest growth defect in the Δ*atcS* strain. *T. denticola* can use carbohydrates but primarily ferments amino acids as their energy and carbon source. Reduced dentilisin activity in the Δ*atcS* strain may produce fewer peptides and free amino acids for metabolism, which causes the minor growth defect we observed. Additionally, an analysis of the RNA-seq data reveals an abundance of genes involved in glycine metabolism are downregulated (Table S1). While glycine is not essential for *T. denticola*, it can be used as a carbon source for one-carbon metabolism or to synthesize other amino acids via the glycine cleavage system. Glycine can also be used to generate ATP via the glycine reductase complex. Both glycine catabolic pathways are repressed in the Δ*atcS* strain relative to ATCC 35405. Future studies may explore the role of AtcS/R in regulating *T. denticola* central metabolism. We are currently developing an *atcR* overexpressing strain and a conditional knockout of *atcR* that, in combination with chromatin immunoprecipitation approaches, may be used in future studies to further inform the role of AtcS/R in the differential expression of genes reported here.

As mentioned above, we previously predicted that AtcR would regulate *TDE2770* (*fliK*) expression, and this study demonstrated that *fliK* was significantly downregulated in the Δ*atcS* mutant (28). In addition to *fliK*, the expression of several other motility-associated genes was reduced. Functionally, we identified a significant reduction in the motility of Δ*atcS*. Motility allows *T. denticola* to navigate the oral cavity’s viscous environment and penetrate the gingival crevices’ depths, allowing for the colonization of an ideal anaerobic environment for survival. Motility aids in *T. denticola*’s ability to integrate into multi-species biofilms on tooth surfaces and contributes to synergistic interactions with *P. gingivalis* (49, 50). The rapid, corkscrew-like motility allows *T. denticola* to evade phagocytosis by immune cells (51). Motility also enables *T. denticola* to invade epithelial cells and host tissues, directly contributing to tissue damage and inflammation through bacterial factors (i.e., Msp, dentilisin, hydrogen sulfide production), and indirectly by stimulating the host’s immune response (39). Finally, motility allows *T. denticola* to move toward nutrient sources, such as peptides and amino acids, essential for its growth and metabolism. This chemotactic movement ensures the bacterium can thrive in nutrient-poor environments. We demonstrated reduced chemotactic responses of the Δ*atcS* strain. Still, these results were more likely due to the defect in motility than a specific defect in recognition and response to glucose or hemin as chemoattractants.

We demonstrated that the deletion of *atcS* reduces dentilisin activity, which also impacts interactions with host cells (16, 52). Dentilisin, a surface protease complex, plays a key role in periodontal disease by degrading extracellular matrix components like fibronectin and laminin, facilitating bacterial movement through host tissues. Additionally, dentilisin disrupts immune responses by cleaving cytokines and complement proteins (i.e., factor H, C3, and C4), weakening the host’s defenses (32–34). It also promotes inflammation by activating toll-like receptor 2 (TLR2) pathways and modulating host cell signaling to sustain chronic infection (41). The Δ*atcS* strain had reduced attachment to gingival keratinocytes. While the molecular mechanism behind this is unclear, it is unlikely to be explained by reduced dentilisin abundance and activity, as the CCE strain, which completely lacks PrtP and SAAPFNA hydrolysis, displayed a more modest reduction in attachment to TIGKs. Again, while motility is reduced, the *T. denticola* flagella are periplasmic, thus not responsible for interactions with host cells. Aside from the role of AtcS, it is striking how modest the impact of dentilisin had on CCE attachment to host cells compared to ATCC 35405, but the invasion of TIGKs was severely attenuated. We also expected a more pronounced induction of apoptosis by *T. denticola* ATCC 35405 than we observed. This may be a difference in host cell models. Leung et al. observed ~80% apoptosis in HeLa, CHO, and human gingival fibroblasts; however, periodontal ligament cells were resistant to *T. denticola*-induced apoptosis (53). Here, we observed ~20% of TIGK cells were late apoptotic/necrotic following *T. denticola* infection, and our previous transcriptomic analysis did not detect induction of pro-apoptotic genes or pathways following *T. denticola* infection of HIGK cells (17). Another significant difference is that we treated gingival epithelial cells with an MOI of 100, whereas previous studies have used an MOI between 1,000 and 8,000 to induce apoptosis (53). Regardless, we did not observe either CCE or the Δ*atcS* strains impacting early to late apoptosis.

Historically, gene deletions in *T. denticola* are rarely restored by complementation due to limitations in genetic systems. Recent advances by Johnston et al. provided a synthetic, stably replicating plasmid to the genetic toolbox for ATCC 35405 (48). While working to complement the *atcS* deletion for future studies, we confirmed the impact of the *atcS* gene deletion using four independently constructed Δ*atcS* clones (Fig. S1, S3, and S4). We also performed genome sequencing, identifying all single-nucleotide polymorphisms (SNPs) or genomic variants between the ATCC 35405 and Δ*atcS* strains (Table S2). These analyses strongly suggest that the phenotypes described in this study resulted from the specific deletion of *atcS*.

The mechanisms *T. denticola* uses to sense and respond to environmental changes have yet to be discovered. This study provides a more complete understanding of the role of AtcS in the physiology and pathogenicity of *T. denticola*. Still, significant gaps in knowledge remain. Future studies are underway to determine how AtcS activity is regulated, comprehensively describe the role of AtcR, and determine how AtcS/R impacts other signal transduction systems. *T. denticola* also exists in a polymicrobial environment, and we plan to explore the role of AtcS/R in biofilm development and polymicrobial interactions.

## MATERIALS AND METHODS

### Bacterial strains, gingival keratinocytes, and culture methods

*T. denticola* strains ATCC 35405 were grown in NOS medium under anaerobic conditions (5% H_2_, 5% CO_2_, 90% N_2_) at 37°C. For growth curve studies, *T. denticola* strains were grown in NOS, and the absorbance (600 nm) was measured daily for 5 days. Growth studies were performed with three independent experiments with three technical replicate measurements recorded per day. TIGKs were maintained at 37°C and 5% CO_2_ in DermaLife K serum-free complete medium (Lifeline Cell Technologies) (40). TIGK cells were seeded in 6-well plates, grown to 70%–90% confluency before use in each assay described below, and counted using a Countess FL2 cell counter (Thermo).

### Construction of the D*atcS* strain

To generate an isogenic deletion of *atcS* (*tde0032*), a gene was synthesized by GenScript and cloned into pUC57 containing the *ermB* cassette flanked by a 500 bp region upstream and downstream of *atcS*. The *ermB* sequence was derived from the *ermF-ermB* cassette used in prior studies (54). No exogenous promoter was used in this construct, and the expression of *ermB* will be driven by the native promoter upstream of *tde0034*. The resulting plasmid (pGS15) was used as a template for PCR to amplify the knockout construct. As previously described, the purified PCR product was electroporated into chemically competent *T. denticola* ATCC 35405 cells (55). Briefly, 5 mg of purified PCR product was added to 50 mL of electrocompetent *T. denticola* cells in electroporation cuvettes with a 2 mm gap and immediately transformed with a single 2.5 kV pulse. Cells were immediately placed in 1 mL of pre-reduced NOS for 1 day and diluted with 4 mL of pre-reduced NOS after 24 hours of recovery. The bacteria were then subsurface plated in NOS containing 1.5% Noble agar and 0.5 mg/mL clindamycin to select for transformants, and incubated in anaerobic jars at 37°C. In our experience, *T. denticola* rapidly develops spontaneous resistance to erythromycin but not to clindamycin; thus, it is our selection of choice. Colonies appeared 10–14 days after plating and were removed from the plates with wide-bore pipette tips and grown in NOS containing 0.5 µg/mL clindamycin for screening by PCR, as described below.

### Genome analysis

*T. denticola* ATCC 35405 and the D*atcS* mutant were grown in NOS for 4 days before collecting the cells by centrifugation (5,000 × *g* for 5 minutes), and genomic DNA was purified using the Wizard gDNA Purification Kit (Promega). Azenta Life Sciences sequenced the purified gDNA using paired-end 2 × 150 Illumina sequencing on a fee-for-service basis. Sequencing adaptors and low-quality reads were trimmed, and the cleaned reads were aligned to the *T. denticola* reference genome (GCA_000008185.1) using Geneious Prime (v2025.0). Both 35405 and D*atcS* had 99.6% of the cleaned reads (>13 M reads for both strains) aligned to the genome, which resulted in 10× coverage of the genome for both strains. Variants and SNPs were identified, and visualizations were again generated using Geneious Prime (Fig. S1; Table S2).

### PCR and quantitative reverse transcription (qRT)-PCR

The presence or absence of *T. denticola* genes was assessed by PCR. Genomic DNA was purified using the Wizard Genomic DNA purification kit (Promega), which was used as the template for PCR using gene-specific primers and Phusion polymerase (New England Biolabs). Products were analyzed by separation on an agarose gel, stained with SYBR Safe (Thermo Scientific), and visualized using a ChemiDoc (Bio-Rad). QRT-PCR was performed to assess gene expression. RNA was isolated using TRIzol reagent and phase separation, and RNA concentrations were quantified using the NanoDrop One system (Thermo Scientific). DNA was removed using the DNA-free DNA Removal kit (Thermo Scientific), and cDNA was synthesized from total RNA (2 µg RNA per reaction) using a high-capacity cDNA reverse transcription (RT) kit (Applied Biosystems). As a control for gDNA contamination, RNA was also mock treated without reverse transcriptase (no RT control). Quantitative RT-PCR (qRT-PCR) was performed with 10 ng cDNA and Power SYBR Green PCR master mix with gene-specific primers using an Applied Biosystems QuantStudio 3 system. The cycle threshold (*C_T_*) values were determined, and mRNA expression levels were normalized to 16S rRNA expression and expressed relative to the values for ATCC 35405 according to the 2^−ΔΔ*CT*^ method.

### Transcriptomics

*T. denticola* ATCC 35405 and the D*atcS* mutant were grown in NOS for 4 days before collecting the cells by centrifugation (5,000 × *g* for 5 mintes), and RNA was purified by TRIzol phase separation. RNA samples were isolated from five biological replicates for each strain. Total RNA was provided to the VCU Genomics Core, where quantity and RNA integrity were determined using an Agilent Bioanalyzer RNA Pico assay. Ribosomal depletion was then performed using Illumina’s Ribo-Zero rRNA Removal Kit. The resulting depleted RNA was assessed using an Agilent Bioanalyzer RNA Pico assay to confirm efficient rRNA removal. RNA-seq library construction was performed using the Illumina Stranded Total RNA prep and was sequenced using the Illumina NextSeq 1000/2000P1 reagents (300 cycles). After quality control, trimming, and merging of paired sequence reads using FastQC (56), cutadapt (57), and FLASH (58), respectively, reads were mapped to *T. denticola* genes using Bowtie 2 (59) against the *T. denticola* reference transcriptome (assembly ASM818v1) downloaded from the NCBI GenBank database. Gene expression profiles were normalized by a variance-stabilizing transformation using the variance stabilizing transformation function, and the dissimilarity of the profiles was visualized with the plotPCA function in the DESeq2 package (60) in R software. Differentially expressed genes were tested using the DESeq function in the DESeq2 package. Raw and processed data and metadata are available through the Gene Expression Omnibus (GEO; GSE277225).

### Motility assay

To compare the motility of the *atcS* mutant with the wild type, a swarming assay with semisolid NOS agar (0.5% Noble agar) was used based on previously published motility assays (37). Briefly, the NOS plates were pre-reduced overnight in the anaerobic chamber. All *T. denticola* strains were grown to exponential phase in NOS, centrifuged (5,000 × *g*, 5 minutes), and gently suspended in fresh NOS media. The bacterial suspension (2 µL; 10^7^ cells) was gently pipetted into the soft agar plates, ensuring all the suspension were below the surface. Plates were incubated under anaerobic conditions for 4 or 8 days, and the diameter of bacterial plaques was measured in millimeters. Each strain’s motility was assessed in three independent experiments with two technical replicates per experiment.

### Chemotaxis assay

Capillary tube assays were adapted to the anaerobic conditions required for *T. denticola* 35405 wild type and the *atcS* mutant. Approximately 10^8^ cells/mL were suspended in chemotaxis buffer (0.15 M NaCl, 10 mM NaH_2_PO_4_, pH 7.6) containing 0.5% methylcellulose to enhance *T. denticola* translocation and were assessed for chemotactic responses to glucose and hemin (37). Cell suspensions were aliquoted into a 96-well plate and covered with adhesive film. Capillary tubes (1.15 mm internal diameter) were filled with either glucose (0.1 or 1.0 mM) or hemin (0.25 or 0.5 µg/mL) in chemotaxis buffer, and inserted into the cell suspension. Capillary tubes filled with chemotaxis buffer were used as a background control. After 3 hours of anaerobic incubation at 37°C, the contents of the capillary tubes were expelled into the microcentrifuge tubes. The Petroff–Hausser bacterial counting chamber was used for cell enumeration via dark-field microscopy. The average number of spirochetes per field was counted by averaging cell counts from 10 fields of view. The error bars represent the standard error of three independent experiments with three technical replicates.

### SAAPFNA hydrolysis for dentilisin activity

The proteolytic activity of dentilisin was measured through the hydrolysis of the chromogenic substrate SAAPFNA by *T. denticola* wild-type and mutant strains. *T. denticola* strains were grown to exponential growth phase prior to assay. The optical density (OD) of each strain were measured and set to an OD of 0.5 in 1 mL of assay buffer (50 mM Tris-HCl, 100 mM NaCl, 1 mM CaCl_2_, pH 8.0). Assay buffer (50 mL) or different strains were aliquoted to a 96-well plate, and 50 mL of SAAPFNA reconstituted in assay buffer was added to each well. The protease activity of each strain was assayed in the iD3 SpectraMax on 405 nm absorbance kinetic setting for 45 minutes with 5 minute intervals at 37°C. Average absorbance levels were plotted relative to the SAAPFNA activity of strain 35405. Error bars represent the standard error of three independent experiments with five technical replicates.

### Flow cytometry for attachment and invasion of TIGK cells

*T. denticola* ATCC 35405, Δ*atcS*, and CCE were adjusted to 0.2 OD_600_ (3.5 × 10^8^ cells/mL) and labeled with CellBrite-488 fluorescent membrane stain (Biotium). Labeled bacteria were then added to TIGKs seeded in 6-well plates and incubated for either 30 or 120 minutes under 5% CO_2_ at 37°C. Following incubation, cells were washed twice with sterile PBS, detached with trypsin, and resuspended in PBS. Data were acquired on a BD FACS Canto II to assess attachment to TIGKs, and gated single cells were analyzed for percentage of CellBrite-488 with FlowJo software. Samples were acquired on a Cytek Amnis Imagestream MKII to assess *T. denticola* attachment and invasion into TIGKs, compensating with single-stain controls. These studies were performed in four independent experiments, each analyzing ~10,000 cells. Here, *T. denticola* was stained as described above, and the TIGK cell membrane was also stained with Cellbright-640 membrane stain (Biotium). For experimental samples, a total of 2,000 cells were collected. The area of the brightfield-defined cells as an indicator of size versus the aspect ratio of the brightfield parameter as an indicator of roundness to ensure sufficient single-cell populations were collected. In-focus cells were gated according to the Gradient RMS of the brightfield parameter. Data were analyzed using the Image Data Exploration and Analysis Software (IDEAS) version 6.0 by Amnis. Bacteria were identified using a spot count mask based on the Cellbright488 parameter, while a combined tight object mask identified the TIGKs off of the Cellbright-640 parameter and the brightfield. The association of bacteria and the co-cultured cells was determined by the interaction of the bacteria and cell masks, as measured by the external portion of the cell mask for attachment binding or the internal portion of the cell mask for internalization. The percentage attached or internalized was determined as a ratio of cells containing one or more bacteria attached or internalized to total in-focus cells.

### Assessment of apoptosis by flow cytometry

The early versus the later stage of apoptosis was analyzed using the Annexin V Conjugates for Apoptosis Detection kit (Invitrogen) and propidium iodide (PI) staining. TIGK cells seeded in 6-well plates were infected with *T. denticola* ATCC 35405, Δ*atcS*, or CCE at an MOI of 100 for 18 hours. Uninfected TIGKs were a control for healthy cells. Cells were harvested by centrifugation (1,000 × *g* for 10  minutes), resuspended in annexin-binding buffer (10 mM HEPES, 140 mM NaCl, and 2.5 mM CaCl_2_, pH 7.4), and incubated with the Annexin V-allophycocyanin (APC) and PI in the dark for 15 minutes at room temperature. Afterward, the stained cells were quantified by flow cytometry using a BD FACS Canto II with 488 nm excitation for PI (emission collected at 530 nm) and 633 nm excitation for Annexin V-APC (emission collected at 660 nm), and analyzed using FlowJo software. Data are representative of two independent experiments, each quantifying ~10,000 cells.

### Murine alveolar bone loss

BALB/c mice, 8 to 10 weeks old, were obtained from Charles River Laboratory. Ten female mice were used per group, as studies have shown no gender-dependent differences in alveolar bone loss in a periodontal disease model (61). Mice were fed a standard diet with water *ad libitum*. *T. denticola* ATCC 35405 and Δ*atcS* strains (10^9^ CFU) were resuspended in 0.1 mL of PBS with 2% CMC and orally inoculated into mice over 12 days at 2 day intervals. A vehicle control group was inoculated with 2% CMC in PBS alone. Forty-two days after the last infection, the mice were euthanized. Skulls were fixed with 10% neutral buffered formalin and analyzed by microcomputed tomography (μCT) using a SkyScan 1076 micro-CT scanner (Bruker, Billerica, MA, USA). The following parameters for scanning were used: a resolution of 7.9 µm, a rotation step of 0.2°, averaging frames 2, a source voltage of 94 kV, and a source current of 85 µA. The right and left mandibles and maxillae were examined, assessing the distance from the CEJ to the alveolar bone crest (ABC) at 16 points per molar. All values were averaged and reported for the 10 mice per group with standard error of the mean (SEM) and were analyzed by one-way analysis of variance (ANOVA) and Tukey’s post hoc test.

### Statistical analyses

GraphPad Prism 10.3.1 software was used for statistical analyses, and data were evaluated by a *t*-test or a one-way ANOVA with Tukey’s or Dunnett’s multiple-comparison test, as described in the figure legend for each experiment.

## SUPPLEMENTAL MATERIAL

Supplemental figures iai.00112-25-s0001.pdf Fig. S1 to S5.

Table S1 iai.00112-25-s0002.xlsx The differential expression of all genes in the atcS deletion strain compared to ATCC 35405.

Table S2 iai.00112-25-s0003.xlsx Genomic variants identified in the atcS mutant based on WGS.

ASM does not own the copyrights to Supplemental Material that may be linked to, or accessed through, an article. The authors have granted ASM a non-exclusive, world-wide license to publish the Supplemental Material files. Please contact the corresponding author directly for reuse.
