# Supplementary material for: The unique histidine kinase, AtcS, regulates motility and pathogenicity of the periodontal pathobiont, Treponema denticola
Source: Infect Immun. 2025 Apr 2;93(5):e00112-25. doi: 10.1128/iai.00112-25 (PMC12070737; doi:10.1128/iai.00112-25)
Supplement: Supplemental figures — Fig. S1 to S5. [file iai.00112-25-s0001.pdf]

Supplemental Figures and Legends

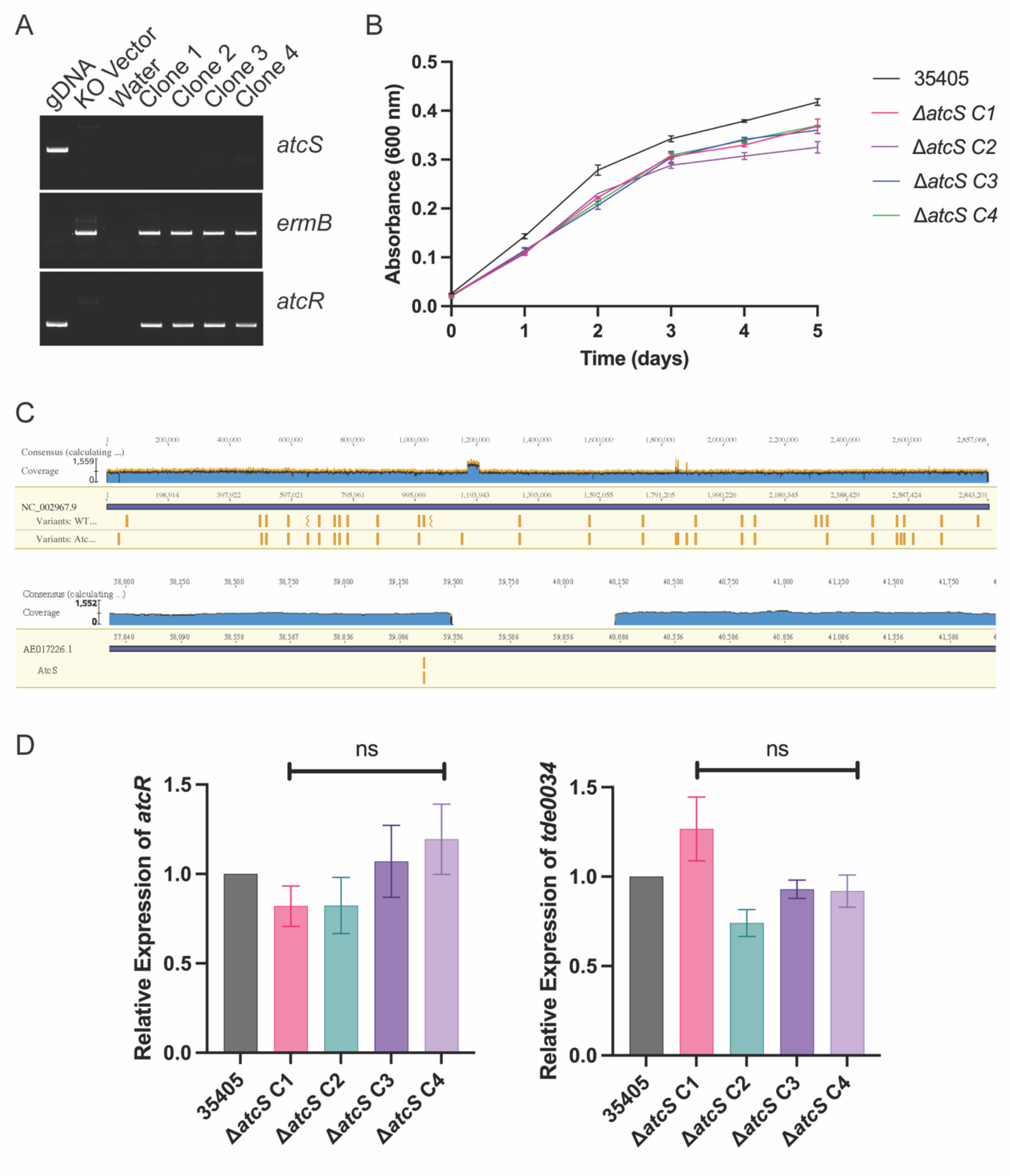

Supplemental Figure 1. Analysis of four independent clones of Δ*atcS*. (A) The *atcS* gene was replaced by *ermB* by allelic exchange, and we isolated four unique clonal isolates confirmed by PCR using primers specific to *atcS* and *ermB*. We confirmed the deletion of

atcS did not impact the presence of the upstream *atcR* gene. **(B)** We monitored the growth of all Δ*atcS* clones compared to the parental ATCC 35405 by measuring absorbance at 600 nm daily. Data are the average of 3 independent cultures with standard error of the mean (3 replicate measurements per time-point). **(C)** We then performed whole genome sequencing of the Δ*atcS* clone used throughout this study (clone 2) and compared the genome to the parental ATCC 35405. Both sequences were compared to the reference genome. The Δ*atcS* strain had no reads that mapped between 39,321 and 40,067 bp, confirming the *ermB* cassette replaced the *atcS* gene exactly where it was intended. **(D)** ATCC 35405 and the 4 clones of Δ*atcS* were grown in NOS for 4 days before total RNA was collected and qRT-PCR assessed gene expression. While not shown, no RT controls revealed no gDNA contamination. These data demonstrate the deletion of *atcS* does not impact the expression of the neighboring *atcR* or *tde0043* genes. Data are the average of 3 biological replicates with SEM. Data were analyzed by one-way ANOVA and Dunnett's post *hoc test* (*p<0.05, **p<0.01, ***p<0.001, ****p<0.0001).

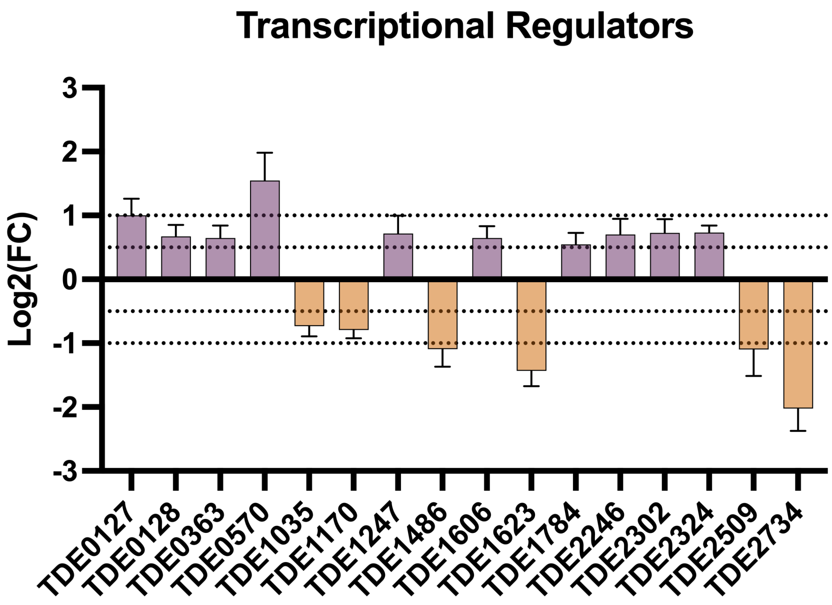

Supplemental Figure 2. Deletion of *atcS* impacts the expression of many putative transcriptional regulators. A bar graph of differential expression with the standard error for genes identified in the RNA-seq associated with transcriptional regulation. Genes significantly repressed in Δ*atcS* relative to ATCC 35405 are orange, while induced genes are purple. The dotted lines represent the log2FC cutoffs for both the relaxed and stringent analysis.

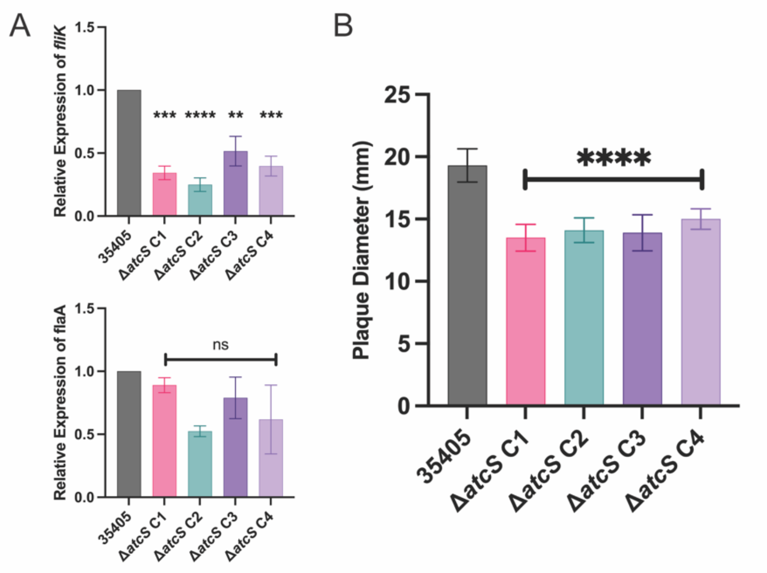

Supplemental Figure 3. All Δ*atcS* clones have reduced fliK expression and reduced swarming motility. (A) ATCC 35405 and the 4 clones of Δ*atcS* were grown in NOS for 4 days before total RNA was collected and qRT-PCR assessed gene expression. While not shown, no RT controls revealed no gDNA contamination. Consistent with the RNA-seq, deletion of *atcS* impacts *fliK* expression but does not significantly impact *flaA* expression. Data are the average of 3 biological replicates with SEM. Data were analyzed by one-way ANOVA and Dunnett's *post hoc test* (**p<0.01, ***p<0.001, ****p<0.0001). **(B)** Wild type (35405) and the Δ*atcS* clones were inoculated into NOS plates solidified with Noble agar, and swarming motility was monitored by measuring the plaque diameter after 8 days of incubation. Data are the average of 3 independent experiments with standard deviation and were analyzed using the one-way ANOVA with Dunnett's *post hoc* test (****p<0.0001).

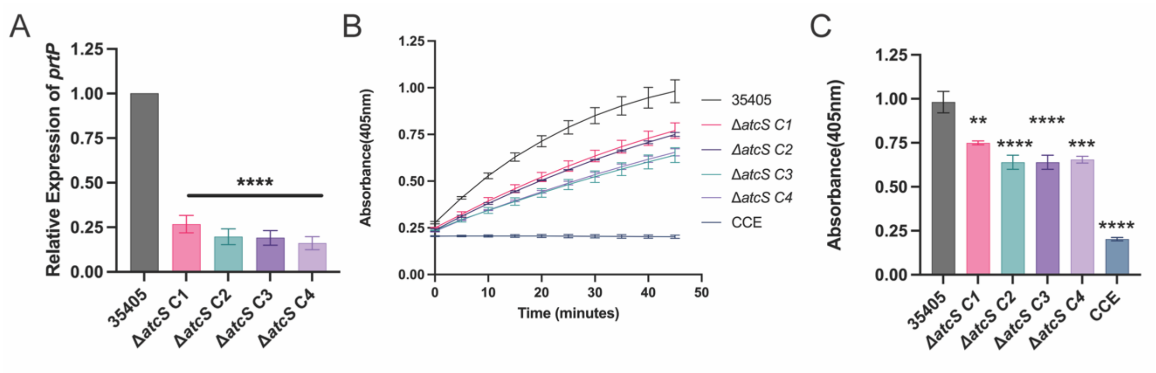

Supplemental Figure 4. All Δ*atcS* clones have reduced *prtP* expression and attenuated dentilisin activity. (A) ATCC 35405 and the four clones of Δ*atcS* were grown in NOS for 4 days before total RNA was collected and qRT-PCR assessed gene expression. While not shown, no RT controls revealed no gDNA contamination. Consistent with the RNA-seq, deletion of *atcS* impacts the expression of *prtP*. Data are the average of 3 biological replicates with SEM. Data were analyzed by one-way ANOVA and Dunnett's *post hoc test* (****p<0.0001). **(B)** The dentilisin activity of each strain was assessed by the hydrolysis of the SAAPFNA substrate colorimetrically by measuring the absorbance at 405 nm every 5 min for 45 min. CCE was used as a negative control. Results are the average of 3 replicates with standard deviation. **(C)** The final absorbance after 45 min is graphed. Data are the average of 3 independent experiments with standard deviation and were analyzed using the one-way ANOVA with Dunnett's *post hoc* test (**p<0.01, ***p<0.001, ****p<0.0001). These data demonstrate all Δ*atcS* clones have reduced dentilisin activity compared to ATCC 35405.

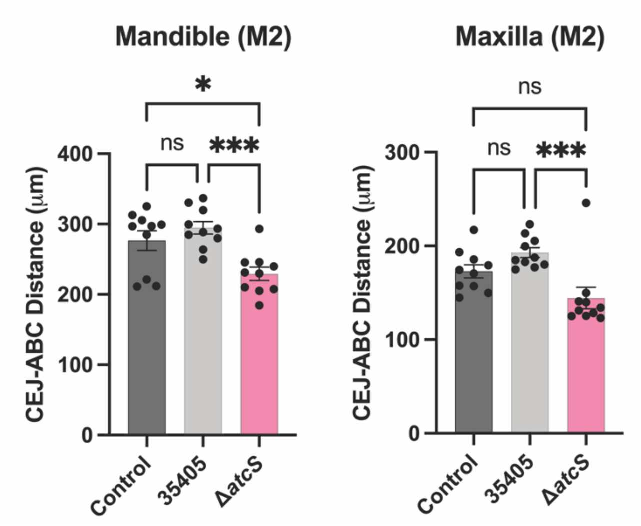

Supplemental Figure 5. Δ*atcS* causes reduced alveolar bone loss that ATCC 35405. Female Balb/C mice were inoculated with either ATCC 35405 (black), Δ*atcS* (pink), or vehicle control (grey) (n=10 animals per group). Following a 6-week incubation, alveolar bone loss was assessed at the second maxillary and mandibular molar using μCT. Data are the average distance (μm) between the cementoenamel junction (CEJ) and the alveolar bone crest (ABC) for each mouse and the standard error of the mean. Data were analyzed by one-way ANOVA with Tukey's *post-hoc* test (not significant (ns), *p<0.05, ***p<0.001).
